# Effects of Dietary Selenium on Histopathological Changes and T Cells of Spleen in Broilers Exposed to Aflatoxin B_1_

**DOI:** 10.3390/ijerph110201904

**Published:** 2014-02-10

**Authors:** Kejie Chen, Xi Peng, Jing Fang, Hengmin Cui, Zhicai Zuo, Junliang Deng, Zhengli Chen, Yi Geng, Weimin Lai, Li Tang, Qingqiu Yang

**Affiliations:** Department of Veterinary Medicine, Sichuan Agricultural University, Ya’an 625014, China; E-Mails: ckj930@126.com (K.C.); pengxi197313@163.com (X.P.); zzcjl@126.com (Z.Z.); dengjl213@126.com (J.D.); chzhli75@163.com (Z.C.); gengyisicau@126.com (Y.G.); nwm_mm2004@163.com (W.L.); tangyimingtt@163.com (L.T.); magician-rookies@hotmail.com (Q.Y.)

**Keywords:** sodium selenite, aflatoxin b_1_, histological lesion, T-cell subsets, flow cytometry, broilers

## Abstract

Aflatoxin B_1_ (AFB_1_), which causes hepatocellular carcinoma and immune-suppression, is commonly found in feedstuffs. To evaluate the ability of selenium (Se) to counteract the deleterious effects of AFB_1_, two hundred 1-day-old male avian broilers, divided into five groups, were fed with basal diet (control group), 0.3 mg/kg AFB_1_ (AFB_1_ group), 0.3 mg/kg AFB_1_+0.2 mg/kg Se (+Se group I), 0.3 mg/kg AFB_1_+0.4 mg/kg Se (+Se group II) and 0.3 mg/kg AFB_1_+0.6 mg/kg Se (+Se group III), respectively. Compared with control group, the relative weight of spleen in the AFB_1_ group was decreased at 21 days of age. The relative weight of spleen in the three +Se groups was higher than that in the AFB_1_ group. By pathological observation, the major spleen lesions included congestion in red pulp and vacuoles appeared in the lymphatic nodules and periarterial lymphatic sheath in the AFB_1_ group. In +Se groups II and III, the incidence of major splenic lesions was decreased. The percentages of CD_3_^+^, CD_3_^+^CD_4_^+^ and CD_3_^+^CD_8_^+^ T cells in the AFB_1_ group were lower than those in control group from 7 to 21 days of age, while there was a marked increase in the three +Se groups compared to the AFB_1_ group. The results indicated that sodium selenite could improve the cellular immune function impaired by AFB_1_ through increasing the relative weight of spleen and percentages of splenic T cell subsets, and alleviating histopathological spleen damage.

## 1. Introduction

Aflatoxins are one type of mycotoxin, which are fungal secondary metabolites in food. Among the identified aflatoxins, aflatoxin B_1_ (AFB_1_) is the predominant form, presents the highest carcinogenic effects, and is classified as a Group I human carcinogen by the International Agency for Research on Cancer [1]. Doses as low as 15-30 µg/kg can cause responses in poultry, known to be extremely sensitive to the toxic effects of AFB_1_ [2]. Besides carcinogenic effects, acute or chronic aflatoxicosis in poultry birds results in decreased meat/egg production, immunosuppression [3,4], and increased susceptibility to disease [5]. Several approaches have indicated that many adsorbents are capable of binding aflatoxins and preventing or reducing their detrimental effects on animals [6].

Selenium (Se), as an essential trace nutrient for animals and humans, has multiple roles in biological systems. The importance of selenium in the optimal functioning of the immune system has been well established. Se incorporates into immune-important organs, such as the spleen and lymph nodes [7], and selenium compounds regulate the function of neutrophils, NK cells, B lymphocytes, and T cells [8]. Moreover, Se indeed plays an important role in cancer prevention [9].

The spleen represents the largest lymphoid tissue, and it is widely considered to be of vital importance in the whole immune function of the body [10]. The percentage of splenic T cell subsets is an important parameter which represents the composition of mature T cells in the body, which decides the biological function of mature T cells and finally relates to the cellular immune function of the body. Previous studies have revealed that AFB_1_ causes spleen tissue damage [11], induces mutations of splenic lymphocytes [12], and decreases the number of CD_4_^+^ and CD_8_^+^ T cells in rat [13]. However, Se supplementation enhanced the proliferation and differentiation of CD_4_^+^ [14] and CD_8_^+^ T cells [15].

Despite the fact that Se has been reported to improve the immune response in broilers fed with aflatoxin contaminated diets [16], the effects of Se against AFB_1_-induced splenocyte damage have rarely been reported. In the present research, experiments were conducted to examine the effects of dietary sodium selenite on AFB_1_-induced changes of relative weight, histological lesions and T lymphocyte subsets of spleen, which reflect the immune function of chickens. The results could provide helpful insightss for similar studies in both human and other animals in the future.

## 2. Materials and Methods

### 2.1. Chickens and Diets

Two hundred 1-day-old healthy male avian broilers were obtained from a commercial rearing farm (Wenjiang Poultry Farm, Sichuan Province, China) and divided into five groups fed on diets as follows: control group, AFB_1_ group (0.3 mg/kg AFB_1_), +Se group I (0.3 mg/kg AFB_1_+0.2 mg/kg Se), +Se group II (0.3 mg/kg AFB_1_ + 0.4 mg/kg Se) and +Se group III (0.3 mg/kg AFB_1_ + 0.6mg/kg Se). By hydride-generation atomic absorption spectroscopy, the content of Se in the control group diet was 0.404 mg/kg. Broilers were housed in cages with electrically heated units and were provided with water as well as the aforementioned diets *ad libitum* for 21 days. Nutritional requirements were adequate according to the 1994 National Research Council guidelines [17] and the Chinese Chicken Feeding Standard (NY/T33-2004).

### 2.2. Relative Weight of Spleen

At 7, 14, and 21 days of age during the experiment, after the body weight was measured, five birds in each group was euthanized and necropsied. The spleen was dissected from each chick and weighed after dissecting the connective tissue around the organ. Related weight of spleen was calculated by the following formula:

Related weight = organ weight (g)/body weight (kg)

### 2.3. Pathological Observation

After weighing, spleens were fixed in 4% paraformaldehyde and routinely processed in paraffin. Thin sections (5 μm) of each tissue were sliced from each block and mounted on glass. Slides were stained with hematoxylin and eosin Y. Histological slides were examined on an Olympus light microscope (Olympus, Tokyo, Japan). 

### 2.4. Determination of Splenic T-cell Subsets

The spleens of five birds in each group were taken to determine the percentages of CD_3_^+^, CD_3_^+^CD_4_^+^, CD_3_^+^CD_8_^+^ T cells by the flow cytometry method and calculate the CD_4_^+^/CD_8_^+^ ratio at 7, 14, and 21 days of age during the experiment. In the flow cytometry method splenic single cell suspension was prepared by gently cutting each spleen into pieces using dissecting scissors and then filtering through nylon gauze. Splenic single cell suspension was centrifuged at 200 × *g* for 5 min. The supernatant was discarded and lymphocytes were collected. The cell concentration was determined by using the normal counting method of blood cells and then diluted to 1.0 × 10^6^ cells/mL with phosphate-buffered saline (PBS). 100 μL cell suspension was transferred to another centrifuge tube. The cells were respectively stained with 10 μL mouse anti-chicken CD3-SPRD (8200-13, SouthernBiotech, Birmingham, AL, USA), mouse anti-chicken CD4-FITC (8210-02, SouthernBiotech) and mouse anti-chicken CD8a-RPE (8220-09, SouthernBiotech) for 15–20 min at room temperature, and then 2 mL PBS was added and centrifugal elutriation performed once. The supernatant was discarded. The cells were resuspended in 0.5 mL PBS and determined by a BD FACS Calibur flow cytometer (BD Co. Ltd., San Diego, CA, USA).

### 2.5. Statistical Analysis

Statistical analysis was performed with SPSS 16.0 for Windows (SPSS Inc., Chicago, IL, USA). All parameters determined in this study were presented as mean ± standard deviation (X ± SD) of the mean. Statistical analyses were performed using one-way analysis of variance. A probability value *p* <0.05 was considered to be significant.

## 3. Results

### 3.1. Changes of Relative Weight of Spleen

No significant differences were observed among five groups at 7 and 14 days of age. At 21 days of age, the relative weight of spleen in the AFB_1_ group was significantly lower (*p* < 0.01) than that in the control group. Compared with that in AFB_1_ group, the relative spleen weights in the three +Se groups were increased (*p* < 0.05 or *p* < 0.01) at 21 days of age. The relative spleen weight in +Se group I was significantly lower (*p* < 0.05) those that in the +Se groups II and III at 21 days of age (Table 1).

**Table 1:** Relative Weight of Spleen Changes (g/kg).

Group	7 Days	14 Days	21 Days
Control group	0.61 ± 0.06	0.55 ± 0.12	0.73 ± 0.07 ^Be^
AFB_1_ group	0.53 ± 0.06	0.51 ± 0.10	0.57 ± 0.05 ^AcDE^
+Se group I	0.53 ± 0.09	0.63 ± 0.11	0.66 ± 0.06 ^bDE^
+Se group II	0.53 ± 0.08	0.60 ± 0.09	0.80 ± 0.05 ^BC^
+Se group III	0.57 ± 0.07	0.57 ± 0.10	0.81 ± 0.06 ^aBC^

Notes: Data are presented with the means ± standard deviation (n = 5). Letter A, B, C, D and E represent the significant difference (*p* < 0.01) between the group and control group, AFB_1_ group, +Se group I, +Se group II, +Se group III, respectively. Letter a, b, c, d and e represent difference (*p* < 0.05) between the group and control group, AFB_1_ group, +Se group I, +Se group Ⅱ, +Se group Ⅲ, respectively.

### 3.2. Pathological Lesions

**Figure 1:**
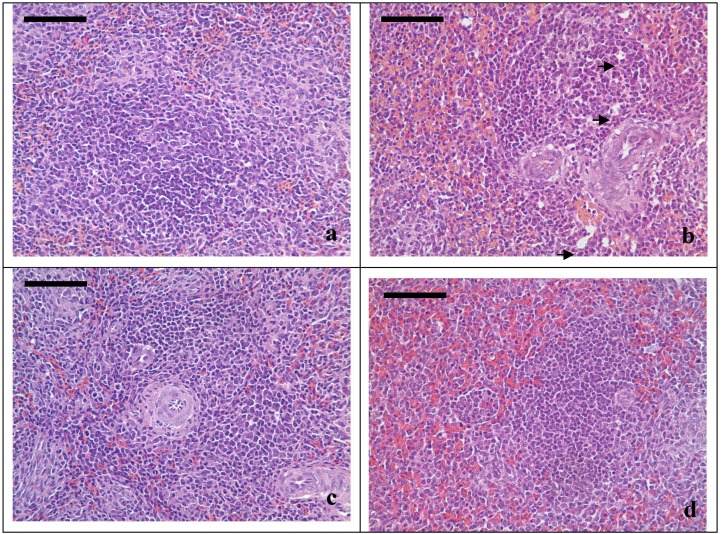
(**a**) Spleen of the 21-day-old chickens in the control group. (**b**) Spleen of a 21-day-old chicken in the AFB_1_ group. In the periarterial lymphatic sheath, the number of lymphocytes was decreased and vacuoles appeared (arrows), congestion in red pulp was observed. (**c**) Spleen of a 21-day-old chicken in the +Se group II. There were no obvious changes compared with control group. (**d**) Spleen of a 21-day-old chicken in the +Se group III. Congestion in red pulp was obvious. *H.E.* bars = 50 μm.

The most typical changes were observed at 21 days of age (Figure 1). Compared with the control group, in the AFB_1_ group, the number of lymphocytes was lightly decreased and vacuoles appeared (arrows) in lymphatic nodule and periarterial lymphatic sheath, as well as congestion in the red pulp. The changes of spleen in the +Se group I were similar to those in the AFB_1_ group but to a lesser degree. There were no marked changes of spleen in +Se group II. The major spleen change in +Se group III was congestion in red pulp. 

The incidence of major splenic lesions is shown in Table 2. At 7 and 14 days of age, the incidence of lesions in the AFB_1_ group and +Se group I was slightly higher than those in the other three groups. At 21 days of age, the incidence of lesions in AFB_1_ group, including congestion in red pulp and appearance of vacuoles, was higher than that in the control group. Compared with the AFB_1_ group, the incidence of lesions in the +Se group II and +Se group III was decreased. The incidences of congestion in +Se group III were higher than those in +Se group II and control group.

### 3.3. Changes of Splenic T-cell Subsets

The percentages of CD_3_^+^, CD_3_^+^CD_4_^+^ and CD_3_^+^CD_8_^+^ T cells in the AFB_1_ group were evidently lower (*p* < 0.01) than those in control group from 7 to 21 days of age. The percentages of CD_3_^+^, CD_3_^+^CD_4_^+^ and CD_3_^+^CD_8_^+^ T cells in the three +Se groups were higher (*p* < 0.05 or *p* < 0.01) than those in the AFB_1_ group from 7 to 21 days of age. At 21 days of age, compared with those in +Se group I, the percentages of CD_3_^+^, CD_3_^+^CD_4_^+^ and CD_3_^+^CD_8_^+^ T cells in +Se group II were markedly increased (*p* < 0.01). The percentages of CD_3_^+^, CD_3_^+^CD_4_^+^ and CD_3_^+^CD_8_^+^ T cells in +Se group III were significantly lower (*p* < 0.01) than those in +Se group II at 21 days of age, as well as the percentage of CD_3_^+^CD_8_^+^ T cells at 14 days of age. There were no remarkable differences among five groups on CD_4_^+^/CD_8_^+^ ratio from 7 to 21 days of age (Table 3).

**Table 2:** Incidence of major spleen lesions.

Time	Pathological Lesions	Control Group	AFB_1_ Group	+Se Group I	+Se Group II	+Se Group III
7 days	Congestion in red pulp	1/5	2/5	1/5	1/5	1/5
	Vacuoles appeared	1/5	1/5	2/5	1/5	1/5
14 days	Congestion in red pulp	1/5	1/5	2/5	1/5	2/5
	Vacuoles appeared	1/5	2/5	1/5	1/5	1/5
21 days	Congestion in red pulp	1/5	4/5	4/5	1/5	3/5
	Vacuoles appeared	1/5	4/5	3/5	1/5	1/5

Note: Incidence of lesions in the spleen among animals from different experimental groups (n = 5).

## 4. Discussion

Spleen, known as the main immune organ, plays an important role in protecting immune response. The relative weight was used to judge the spleen development status. At 21 days of age, the relative weight of spleen in the AFB_1_ group was lower than those in the control group, consistent with the results of Quist *et al.* [18]. Compared with those in the AFB_1_ group, the relative spleen weights in the three +Se groups was increased, which was in agreement with a previous study [19]. The results indicated the AFB_1_ could repress spleen development, while sodium selenite supplemented into the diets could relieve the AFB_1_-induced suppression of spleen development.

**Table 3:** Changes of splenic T cell subsets.

Time	Items	Control Group	AFB_1_ Group	+Se Group I	+Se Group II	+Se Group III
7days	CD_3_^+^ (%)	50.81 ± 4.22B ^CD^	45.55 ± 3.19 ^ACDE^	58.72 ± 3.36 ^ABE^	57.90 ± 3.66 ^ABE^	55.12 ± 3.40 ^BCD^
	CD_3_^+^ CD_4_^+^ (%)	10.11 ± 1.82	6.82 ± 1.83 ^ACDe^	10.60 ± 2.00 ^b^	11.12 ± 2.02 ^B^	10.14 ± 2.02 ^b^
	CD_3_^+^CD_8_^+^ (%)	25.91 ± 2.24 ^BCD^	19.18 ± 3.39 ^ACDE^	28.11 ± 2.33 ^aB^	30.26 ± 2.17 ^ABe^	26.44 ± 2.65 ^Bd^
	CD_4_^+^/CD_8_^+^	0.39 ± 0.04	0.35 ± 0.04	0.37 ± 0.04	0.37 ± 0.04	0.38 ± 0.04
14days	CD_3_^+^ (%)	54.69 ± 1.97 ^BCDE^	41.24 ± 2.45 ^ACDE^	58.84 ± 1.51 ^aBDE^	66.65 ± 1.88 ^ABC^	63.66 ± 1.90 ^ABC^
	CD_3_^+^ CD_4_^+^ (%)	12.19 ± 1.62 ^B^	8.92 ± 1.92 ^ACDe^	12.24 ± 1.73 ^B^	13.40 ± 1.72 ^B^	11.53 ± 1.66 ^b^
	CD_3_^+^CD_8_^+^ (%)	26.02 ± 1.98 ^BD^	14.97 ± 1.77 ^ACDE^	23.60 ± 1.97 ^BD^	31.31 ± 1.81 ^ABCE^	25.71 ± 2.40 ^BD^
	CD_4_^+^/CD_8_^+^	0.44 ± 0.03	0.43 ± 0.06	0.43 ± 0.04	0.42 ± 0.03	0.43 ± 0.04
21days	CD_3_^+^ (%)	57.41 ± 1.89 ^BD^	42.20 ± 1.93 ^ACDE^	59.27 ± 2.14 ^BD^	65.51 ± 2.17 ^ABCE^	60.39 ± 2.02 ^BD^
	CD_3_^+^ CD_4_^+^ (%)	15.34 ± 2.34 ^BD^	8.61 ± 1.62 ^ACDE^	13.97 ± 2.09 ^BD^	18.66 ± 2.03 ^ABCE^	15.12 ± 2.10 ^BD^
	CD_3_^+^CD_8_^+^ (%)	29.27 ± 2.23 ^BD^	16.84 ± 1.99 ^ACDE^	26.54 ± 2.21 ^BD^	35.22 ± 2.04 ^ABCE^	28.92 ± 2.70 ^BD^
	CD_4_^+^/CD_8_^+^	0.52 ± 0.04	0.51 ± 0.04	0.52 ± 0.04	0.53 ± 0.03	0.52 ± 0.04

Notes: Data are presented with the means ± standard deviation (n = 5). Letter A, B, C, D and E represent the significant difference (*p* < 0.01) between the group and control group, AFB_1_ group, +Se group I, +Se group II, +Se group III, respectively. Letter a, b, c, d and e represent difference (*p* < 0.05) between the group and control group, AFB_1_ group, +Se group I, +Se group II, +Se group III, respectively.

Splenic nodules are the place where B lymphocytes gather and mature, and the periarterial lymphatic sheath consists of matured T lymphocytes. By pathological observation, similar to the observations by Omar [20], a decrease of lymphocytes and the appearance of vacuoles in the lymphatic nodules and periarterial lymphatic sheath was observed, indicating suppressed proliferation of B cells and T cells in the AFB_1_ group. Compared with those seen in the AFB_1_ group, the histopathological spleen lesions in the three +Se groups were alleviated. The results showed that 0.2, 0.4, and 0.6 mg/kg Se supplied with the diet could protect splenic lymphocytes from suppression of development induced by AFB_1_, alleviating the adverse effects of AFB_1_ mainly evidenced in the number of lymphocytes, and the humoral and cellular immune functions of the spleen were improved.

T cells migrating from the thymus to the spleen proliferate in the spleen and then migrate to the peripheral blood and lymphatic tissues [21]. T cells can be divided into subsets based on their expression of cell surface proteins. CD3 molecular is the surface marker of mature T cells, most CD_4_^+^ T cells are helper/inflammatory T cells responding to exogenous antigens in association with major histocompatibility complex (MHC) class II molecules and CD_8_^+^ T cells respond to endogenous antigens in association with MHC class I molecules and generally function as cytotoxic T cells [22]. In the present research, the percentages of CD_3_^+^, CD_3_^+^CD_4_^+^ and CD_3_^+^CD_8_^+^ T cells in the AFB_1_ group were lower than those in the control group from 7 to 21 days of age. The results indicated that the immune function of spleen was impaired by AFB_1_.

According to previous research, AFB_1_ induces suppression of thymus development [23]. Our study showed that mature splenic T cells were reduced by dietary AFB_1_. Therefore, the decrease of splenic T cells may be simultaneously due to suppressed development of the thymus and decreased proliferation of T cells in the spleen. It had been confirmed that AFB_1_ could cause selective mitochondrial damage [24,25], disturb the integrity of cell membranes in lymphocytes [26], reduce the proliferation of T cells by decreasing DNA and RNA synthesis [27,28] and induce splenocyte apoptosis [29]. Besides, the congestion in red pulp of spleen was associated with the decreased percentages of CD_3_^+^, CD_3_^+^CD_4_^+^ and CD_3_^+^CD_8_^+^ T cells. It had been reported that hypoxia, caused by congestion, markedly diminished the proliferation of T cells [30,31]. 

On the contrary, the percentages of CD_3_^+^, CD_3_^+^CD_4_^+^ and CD_3_^+^CD_8_^+^ T cells in the three +Se groups were higher than those in the AFB_1_ group from 7 to 21 days. It was shows that appropriate amounts of supplemented dietary Se could increase the percentages of CD_3_^+^, CD_3_^+^CD_4_^+^ and CD_3_^+^CD_8_^+^ T cells inhibited by AFB_1_. According to a previous study, Se could alleviate the destructive oxidative stress caused by AFB_1_ [29]. Moreover, Se, by opposing the effects of AFB_1_, could effectively alleviate the repression of thymus development [23], inhibit AFB_1_-induced DNA damage [32] and promote cell growth [33]. Through histological observation, moreover, the congestion in red pulp could be mitigated, especially in +Se group II. It was shown that the cellular immune function of the body, impaired by AFB_1_, could be improved by an appropriate level of dietary sodium selenite.

Se is an essential nutrient for animals and humans, but high concentrations of Se are toxic when they exceed a threshold [34,35]. In the present study, compared with +Se group II the relative weights of the spleens in +Se group III were similar, but the percentages of CD_3_^+^, CD_3_^+^CD_4_^+^ and CD_3_^+^CD_8_^+^ T cells were significantly decreased. This may result from congestion in red pulp and G_0_/G_1_ arrest of splenocytes induced by excess Se [34]. The results therefore showed that the protective effects of Se were reduced by excess supplementation. 

## 5. Conclusions

According to the results in the present study and the aforementioned discussion, it was concluded that 0.2, 0.4, and 0.6 mg/kg Se supplied in the diets of broilers could alleviate AFB_1_-induced histological lesions, reduced relative weight and decreased T-cell subsets of spleen, and humoral and cellular immune functions could be improved in chickens exposed to AFB_1_. Our study demonstrated that 0.4 mg/kg Se supplied in the diet displayed the best protective effects against 0.3 mg/kg AFB_1_.
